# On the Fallacies Connected with the Iodic Acid Test

**Published:** 1846-10

**Authors:** 


*On the fallacies connected with the Iodic Acid Test..*—Mr. Alfred S. Taylor, in a late number of the *Medical Gazette*, has made some experiments, and arrived at certain results, which, indicating a source of error in standard works on jurisprudence, require to be known. He states, that MM. Devergie and Taufflieb, in testing the stomach of an individual poisoned by sulphuric acid, arrived at a series of conclusions, one of which was to the effect, that on resorting to distillation of the stomach and its contents, certain inconveniences would be avoided by collecting the liberated sulphuric acid, and passing it through iodic acid, which, being decomposed, become a new test for sulphuric acid. M. Taylor proceeds to say:—

"M. Devergie refers to these experiments, and expressly states that the iodic acid, while it is a very delicate test, furnishes a certain proof that sulphuric acid must have existed in the matters submitted to analysis, either in a free state, or in combination with a metallic oxide.

"A case has recently occurred to me, in which the inapplicability of the iodic acid test, under the circumstances in which its use is advised by M. Devergie, will be apparent. Mr. Eastes, surgeon, of Folkstone, lately brought to me for examination the stomach of a man who, it was suspected, had been poisoned by some companions with whom he had been drinking. The contents of the organ were of a dark colour, but the surface of the mucous membrane itself was not carbonized. No trace of sulphuric acid could be detected in the stomach or its contents, by the ordinary process of digestion in water and boiling. The dark grumous matter was found to be nothing more than altered blood, the man having suffered from an attack of hæmatemesis. An opinion was therefore given that the deceased had not died from the effects of sulphuric acid; and this was corroborated by other circumstances which transpired. Had the process advised by MM. Taufflieb and Devergie been employed, there is not the least doubt, from the facts about to be related, that sulphuretted hydrogen would have been obtained, and this, from its action on iodic acid, would have been considered to furnish evidence of the presence of sulphuric acid in the stomach.

"The string with which the stomach was tied appeared somewhat blackened, although quite firm, and free from any signs of corrosion. A portion was dried and heated in a tube to redness; the products of the distillation were received on paper, saturated with starch, and moistened with iodic acid; blue iodide of farina was immediately produced. A portion of clean string, similarly treated, had no effect on the iodic acid. The cause of the difference was undoubtedly owing to the blackened string being saturated with mucus and blood.

"A portion of pure gluten, which contains sulphur, was now employed, and blue iodide of farina was immediately formed. The same result was obtained by heating macaroni, a piece of flannel, dried albumen, dried blood, caoutchouc, and lastly, a portion of the dried human stomach itself, in a case in which it was certain that no sulphuric acid could have been taken, and the organ had been well washed. The fact is, all substances containing sulphur will yield products at a red heat, which easily deoxidize iodic acid; and I would suggest this as a good supplementary test for sulphur in organic matter where the compound of oxide of lead in potash cannot be easily employed.

"It appears from the results of the foregoing experiments that when sulphuric acid cannot be detected in the contents of the stomach in a free state, the chemical investigation must be considered at an end. The medical witness who depends on the results of the dry distillation of the stomach or its contents, may be induced to give a most incorrect opinion.

"I had recommended the employment of the iodic acid test, according to the method above described, for the purpose of detecting sulphuric acid in stains on clothing, accompanying the recommendation with the precaution, that an opinion of the presence of the acid could only be expressed when unstained portions of the cloth similarly treated gave negative results. In a recent examination of some stains on black cloth, I found that the iodic acid was easily decomposed, both by a stained and unstained portion of cloth. The result in the latter case most probably depended on the presence of sulphur in the woollen fabric. It has been already stated, that clean flannel, by dry distillation, easily decomposes iodic acid; but I found that neither linen nor cotton had any action on iodic acid, except when stained with sulphuric acid. Neither of these fabrics contain sulphur; hence, before employing the iodic acid test in these investigations, it would be proper to inquire whether the organic substance be one of those which are known to contain sulphur; if not, the test may be employed, otherwise it should be discarded. No risk can ever be incurred if the analyst only adopts the simple plan of experimenting on a portion of the unstained material in the first instance."—*Ib.*

